# Meeting the Physical and Mental Health Needs of Older Offenders: Does Aging in Place Work in Prison?

**DOI:** 10.1093/geroni/igaa057.064

**Published:** 2020-12-16

**Authors:** Eddy Elmer, Heather Campbell Pope

**Affiliations:** 1 Vrije Universiteit Amsterdam, Vancouver, British Columbia, Canada; 2 Dementia Justice Canada, Kingston, Ontario, Canada

## Abstract

In many countries, the proportion of older people in prison is growing due to longer sentences, increases in convictions for historical offences, and longevity. Moreover, harsh conditions of confinement coupled with the negative effects of a criminal lifestyle may contribute to 'accelerated aging' in this population. Indeed, many prisoners develop health problems that are more commonly seen among people who are up to ten years older. Correctional institutions are increasingly struggling to meet the complex and expensive healthcare needs of these offenders, especially at end-of-life. Some institutions have taken the position that prisons were never intended to be nursing homes, nor can they be adequately adapted to fulfill this role. As a result, these institutions attempt to place some aging offenders in healthcare institutions within the community, provided that their risk to the public can be adequately managed. Other institutions have argued that the needs of aging offenders can be successfully met behind prison walls and have taken steps to allow prisoners to 'age in place.' After summarizing the research on the physical and mental health needs of aging offenders, this presentation considers the advantages and disadvantages of meeting older offenders' healthcare needs both within and outside the prison setting and provides relevant examples of both. Special attention is paid to the issues of social isolation and loneliness: both may contribute to accelerated aging, and perhaps even the risk for re-offending, raising questions about which correctional settings are most beneficial for minimizing these problems.

